# Rapid genotyping of *Toxoplasma gondii* isolates via Nanopore-based multi-locus sequencing

**DOI:** 10.1186/s13568-024-01728-x

**Published:** 2024-06-06

**Authors:** Zisis Koutsogiannis, Paul W. Denny

**Affiliations:** Department of Biosciences, Durham University, Durham, DH1 3LE UK

**Keywords:** *Toxoplasma gondii*, Genotyping, Oxford Nanopore MinION, Multi-locus sequence typing (MLST)

## Abstract

*Toxoplasma gondii* is an obligate intracellular parasite associated with severe disease, especially in the immunosuppressed. It is also a cause of congenital malformation and abortion in both animals and humans and is considered one of the most important foodborne pathogens worldwide with different strains showing variable distribution and differing pathogenicity. Thus, strain-level differentiation of *T. gondii* isolates is an essential asset in the understanding of parasite’s diversity, geographical distribution, epidemiology and health risk. Here, we designed and implemented an Oxford Nanopore MinION protocol to analyse genomic sequence variation including single nucleotide polymorphisms (SNPs) and insertion/deletion polymorphisms (InDel’s) of four different genomic loci, part of protein coding genes *SAG2, SAG3, ROP17* and *ROP21*. This method provided results with the sequencing depth necessary for accurate differentiation of *T. gondii* strains and represents a rapid approach compared to conventional techniques which we further validated against environmental samples isolated from wild wood mice. In summary, multi-locus sequence typing (MLST) of both highly conserved and more polymorphic areas of the genome, provided robust data for strain classification in a platform ready for further adaption for other strains and pathogens.

**Supplementary Information:**

The online version contains supplementary material available at 10.1186/s13568-024-01728-x.

## Key points

- Typing of *T. gondii* is essential for understanding the epidemiology and spread of more virulent strains. 
- Multi-locus sequence typing (MLST) provides several advantages, including accuracy and specificity, over conventional approaches.
- The portable Nanopore-based approach described here represents a cheaper, quicker and less technically demanding approach to MLST in *T. gondii.*

**Graphical Abstract:**

**Supplementary Information:**

The online version contains supplementary material available at 10.1186/s13568-024-01728-x.

## Graphical abstract

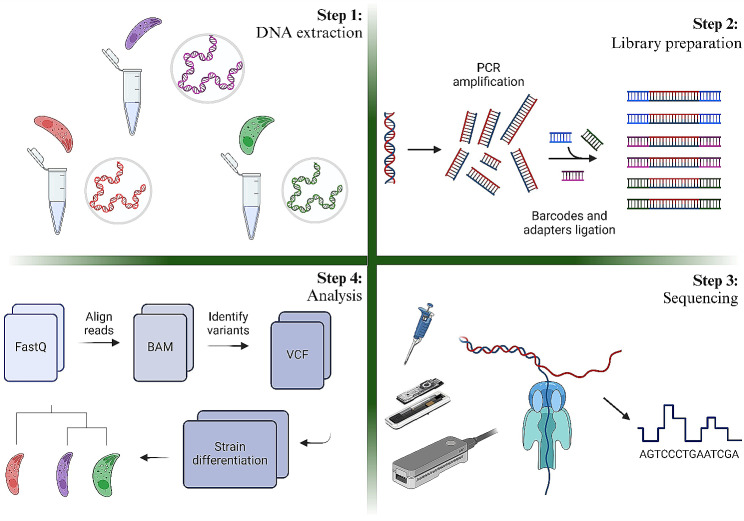

## Introduction

*Toxoplasma gondii* is an obligate intracellular protozoan parasite that belongs to the phylum *Apicomplexa*, a diverse group of single cell eukaryotes that infect humans and animals. *T. gondii* has a complex life cycle with an extensive variety of hosts, although only members of the *Felidae* family (the definite hosts) can support the sexual cycle (Dubey 2021a). Pregnant women and immunocompromised individuals are considered as the primary risk groups. The characteristic features of congenital toxoplasmosis include chorioretinitis, cerebral calcifications, hydrocephalus, microcephaly, abnormal cerebrospinal fluid, hepatosplenomegaly and miscarriages (Jones et al. 2001; McAuley 2014). In immunocompromised individuals re-activation or new infection can cause severe neurological manifestations including life threating encephalitis, chorioretinitis, pneumonia or multiorgan involvement along with acute respiratory failure (Montoya and Liesenfeld 2004).

In addition to major economic losses in livestock (Buzby 1996), *T. gondii* infection poses a significant risk to public health when animals are destined for consumption and via leafy vegetables or fruits contaminated with oocysts shed in the faeces of infected cats (Robert-Gangneux and Darde 2012). Indeed, *T. gondii* is ranked 4th in the global ranking of food-borne parasites (FAO/WHO 2014) and second in the European Union (Bouwknegt et al. 2018). It has been estimated that up to one third of the global population may be infected by *T. gondii* (FAO/WHO 2014; Montoya and Liesenfeld 2004; Petersen and Dubey 2001; Saadatnia and Golkar 2012), and more than a million cases of toxoplasmosis are reported in Europe per annum (WHO 2015).

Despite its wide distribution and sexual reproduction in *Felidae*, the *T. gondii* population does not demonstrate high levels variability due to the primacy of asexual reproduction (Dardé 1996; Sibley and Howe 1996). More than 94% of the total parasite population is categorized into just three biologically discrete clonal lineages referred to as types I, II and III (Grigg and Suzuki 2003). Nevertheless, atypical strains are being regularly reported and these have been associated with severe toxoplasmosis after consumption of imported meat, even in pregnant women with previously acquired immunity (Brito et al. 2023; Elbez-Rubinstein et al. 2009; Hassan et al. 2019; Pomares et al. 2011), and death in the immunocompromised (Stajner et al. 2013). Thus, genetic characterization and strain differentiation is crucial, not only for epidemiological and clinical studies but also the development of effective treatment strategies (de Lima Bessa et al. 2023; Franco et al. 2019).

Conventional genotyping techniques for *T. gondii* involves lengthy procedures including PCR followed by restriction fragment length polymorphism (PCR-RFLP) spread over eight different chromosomes (Su et al. 2006), and micro satellite (MS) analyses with markers from multiple chromosomes (Ajzenberg et al. 2010). Moreover, multi-locus sequence typing (MLST) of specific regions in the parasite genome has been successfully implemented for strain differentiation based on Sanger sequencing (Bertranpetit et al. 2017; Khan et al. 2007), and whole genome sequencing (WGS) has also very recently been deployed for genotyping and in-depth genomic analyses (Joeres et al. 2024; Sundararaman et al. 2024).

Here we present an Oxford Nanopore sequencing-based approach as an alternative method for *T. gondii* genotyping which is significantly quicker and allows for straightforward detection and quantification of rare Single Nucleotide Polymorphisms (SNPs) and Insertions or Deletions (InDel’s) for genotyping. Additionally, it is cheaper and allows for multiple samples to be analysed simultaneously when compared to conventional Sanger sequencing and WGS, in addition, due to the smaller focused data produced, subsequent bioinformatics analyses are quicker and easier. It is also easily utilisable in smaller, less well-resourced laboratories as it does not require expensive large-scale equipment. In the work presented here, we sequenced end-point PCR products from the surface antigen genes *SAG2* (chrVIII: TGGT1_271050; TGME49_271050) and *SAG3* (chrXII: TGGT1_308020; TGME49_308020), in parallel to fragments of the rhoptry genes, *ROP17* (chrVIIb: TGGT1_258580; TGME49_258580) and *ROP21* (chrVIIb: TGGT1_263220; TGME49_263220). The method was validated using two reference strains TGGT1 (Type I) and TGME49 (Type II) before being applied to *T. gondii* genomic DNA (gDNA) isolated from the brains of 4 wood mice (*Apodemus sylvaticus*).

## Materials and methods

### Biomarker selection

Four biomarkers that represent medium to high sequence variation either within (exons) or outside (introns) the coding frame of genes were selected. Three from the literature as outlined in the Results section, another (*ROP21*) based on further comparative analyses of the *ROP* genes in *T. gondii* TGGT1 (Type I) and TGME49 (Type II). For this sequences were retrieved from ToxoDB (toxodb.org) in FASTA format and aligned using EMBOSS Water (www.ebi.ac.uk/jdispatcher/psa/emboss_water).

### *T. gondii* and host cell maintenance

Human foreskin fibroblasts (HFFs; SRC-1041, ATCC, Manassas, Virginia, USA) were cultivated in culture-treated plastics (T-25s) in the presence of Dulbecco’s modified Eagle’s medium (Thermo Fisher Scientific, Waltham, Massachusetts, USA) supplemented with 10% fetal bovine serum (Sigma-Aldrich, St. Louis, Missouri, USA), 2 mM L-glutamine and 1% penicillin-streptomycin. HFF cells were not used beyond passage 20. *T. gondii* TGGT1 (Type I) and TGME49 (Type II) were maintained in vitro by serial passage in monolayers of HFF cells maintained at 37 °C, 5% CO_2_ in a humidified incubator.

### Isolation of *T. gondii* genomic DNA and amplicon production

Genomic DNA (gDNA) was extracted from phosphate saline buffer (PBS) washed *T. gondii* tachyzoites using the QIAamp DNA Blood Mini Kit (Qiagen, Hilden, Germany) as per the manufacturer’s instructions. All PCRs were performed with high fidelity DNA polymerase (New England Biolabs, Ipswich, Massachusetts, USA). All primers used in the assay (Supplemental Table S1) were synthesised by Integrated DNA Technologies (IDT, Coralville, Iowa, USA).

### Sequencing library assembly

Amplicons were pooled together and modified using a Ligation Sequencing gDNA kit (Oxford Nanopore Technologies, Oxford, UK) according to manufacturer’s instructions with modifications. Briefly, 250 fmol of each DNA amplicon was diluted in 50 µL and treated with NEBNext Ultra II End-Prep Enzyme (New England Biolabs) for 20 min at 20 °C to end-prep the DNA (5′ phosphorylated, 3′ dA-tailed), followed by 5 min at 65 °C (Supplemental Table S2). 65 µL of AMPure XP beads (Beckman Coulter, Brea, California, USA) were resuspended in the End-Prep reaction and mixed gently before incubation in a HulaMixer (Thermo Fisher Scientific) for 10 min at room temperature. Beads were washed twice with 250 µL of freshly prepared 70% ethanol and DNA eluted using 27 µL of nuclease free water. 22.5 µL from each end-prepped DNA sample was then barcoded with the Native Barcoding Expansion 1 to 12 (Oxford Nanopore Technologies). Every DNA amplicon was ligated with a unique barcode in a single step reaction (Supplemental Table S3) after treatment with the NEB Blunt/TA Ligase (New England Biolabs) for 20 min at room temperature. Barcoded amplicons were isolated using 65 µL of AMPure XP beads as above. Equimolar amounts of each of the barcoded samples were then mixed and pooled together to a final concentration of 250 fmol and a final volume of 67.5 µL in nuclease free water. Using the NEBNext quick ligation module (New England Biolabs) the DNA barcoded library was ligated to Oxford Nanopore sequencing adapters, the amount optimised (Supplemental Table S4), for 15 min at room temperature. DNA was again isolated using 65 µL of AMPure XP beads but washing with 250 µL of the Short Fragment Buffer (SFB) and eluting with 15 µL of Elution Buffer. The eluate was stored short term in a LoBind tube at 4 °C or used immediately for sequencing on a Flonge cell.

## Priming and loading of the Flonge cell

Flonge cells were stored at 4 °C and used before expiration date as per manufacturer’s instructions to limit sequencing errors and increase sequencing efficiency. Before loading the sequencing library, the Flonge cell nanopores were checked for adequate efficiency. All sequencing experiments were run with at least 80 active nanopores. Flush Buffer and Flush Tether Buffer were loaded onto the Flonge cell, followed by 25 fmol of the prepared sequencing library among with sequencing buffer (SB II) and Loading beads (LB II) as manufacturer’s instruction. Sequencing runs varied from 15 min to 12 h.

### Software and data analysis

All sequencing runs were controlled by minKNOW 23.04.6 (Guppy: 6.5.7; script configuration: 5.5.14; Oxford Nanopore Technologies). Sequencing reads were mapped to *T. gondii *GT1_Genome (ToxoDB-61, toxoDB.org) via EPI2ME 3.6.2 (EP12ME, GitHub) and BAM files were later visualised using IGV_2.16.0 (Integrative Genomics Viewer, GitHub). Quality control analysis of raw reads and BAM files was performed by Nanopore tools/GALAXY version 24.0.rc1 (Oxford Nanopore Technologies).

## Results

### Polymorphisms detection in reference strains

A combination of four different genomic loci were analysed to detect variation between *T. gondii* strains Type I (TGGT1) and Type II (TGME49). Three of these have been previously used for genotyping by PCR-RFLP (the surface antigen typing markers *SAG2* and *SAG3* (Blackston et al. 2001; Gallego et al. 2006; Pena et al. 2013; Rico-Torres et al. 2024; Sabaj et al. 2010; Targa et al. 2023) and the rhoptry virulence marker *ROP17* (Rico-Torres et al. 2023, 2024; Zhang et al. 2014). The other (*ROP21*) has not been yet employed in genotyping studies, however database analyses revealed its potential as new marker. All targets were selected based on their level of variability and, to minimise sequencing times, an amplicon length not exceeding 1500 base pairs (bp). Briefly, the *SAG2* locus presented low to medium variability, while *SAG3*, *ROP17* and *ROP21* loci exhibited medium to high variability. All polymorphisms in the *SAG2, SAG3* and *ROP17* targets were detected within exons, while in *ROP21* polymorphisms were primarily in introns.

**Fig. 1:**
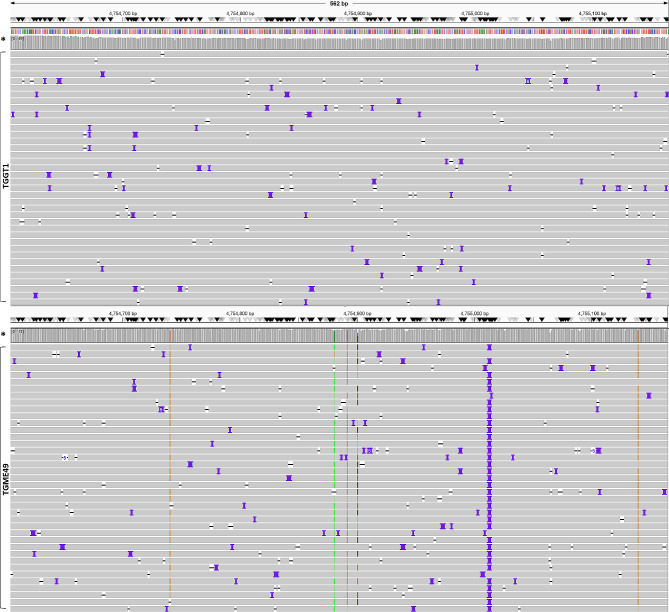
Aligned sequence reads of the SAG2 locus as visualised by IVG. Reads from Type II *T. gondii* (TGME49; bottom panel) were mapped against the Type I *T. gondii* genome (TGGT1; top panel). Five small nucleotide polymorphisms (SNPs) and a three base pair (bp) insertion were detected in the Type II sequence. The analysed fragment length is 560 bp (chrVIII: 4,754,604–4,755,164 [+]). Coverage depth > 500x reads (*). Insertions shown in purple, deletions as dashes. In bottom panel from the left the vertical broken lines represent SNPs in the sense DNA strand as follows: orange change to G (position 138, 289 and 536), green change to A (278), and blue change to C (298). See Table S5

**Fig. 2:**
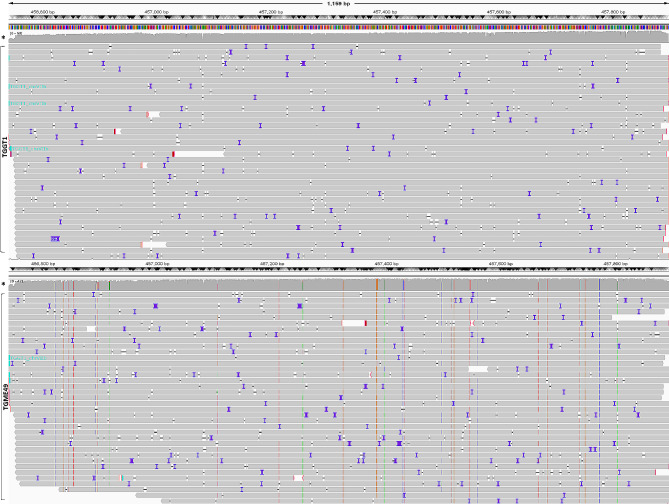
Aligned sequence reads of the *SAG3* locus as visualised by IVG. Reads from Type II *T. gondii* (TGME49; bottom panel) were mapped against the Type I *T. gondii* genome (TGGT1; top panel). 27 SNPs were apparent in the Type II sequence. The analysed fragment length is 1158 bp (chrXII: 456,740–457,897 [−]). Coverage depth > 500x reads (*). In bottom panel from the left the vertical broken lines represent SNPs in the antisense DNA strand as follows: orange change to G (1061, 981, 573, 513, 216, 93), green change to A (1044, 1001, 792, 643, 319, 231, 159), red change to T (1077, 501, 323, 238) and blue change to C (1053, 1005, 685, 514, 468, 466, 401, 351, 150, 125). See Table S6

**Fig. 3:**
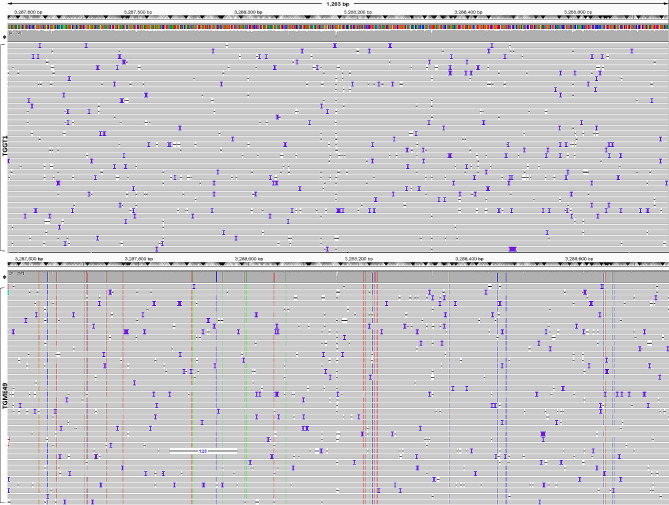
Aligned sequence reads at the *ROP17* locus as visualised by IVG. Reads from Type II *T. gondii* (TGME49; bottom panel) were mapped against the Type I *T. gondii* genome (TGGT1; top panel). 29 SNPs were visible in the Type II sequence. The analysed fragment length is 1280 bp (chrVIIb: 3,287,488–3,288,688 [−]). Coverage depth > 500x reads (*). In bottom panel from the left the vertical broken lines represent SNPs in the antisense DNA strand as follows: orange change to G (1569, 1554, 1486, 1483, 1482, 1447, 1290, 968, 736, 528), green change to A (1417, 1292, 1236, 1196, 1142, 959, 955, 824, 540), red change to T (1247, 1193, 1121, 979, 977, 964) and blue change to C (1573, 721, 544, 524). See Table S7

**Fig. 4:**
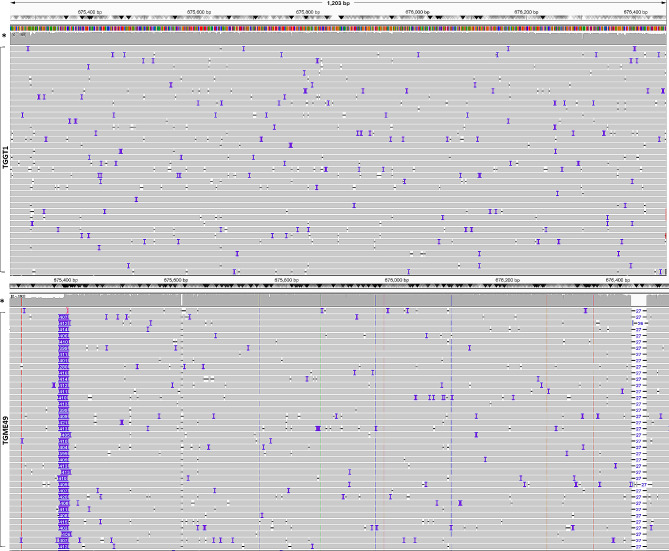
Aligned sequence reads at the *ROP21* locus as visualised by IVG. Reads from Type II *T. gondii* (TGME49; bottom panel) were mapped against the Type I *T. gondii* genome (TGGT1; top panel). Eight SNPs, a 406 bp insertion, and two deletions of two and 27 nucleotides respectively are visible in the Type II sequence. The analysed fragment length is 1441 bp (chrVIIb: 675,115–676,556 [+]). Coverage depth > 500x reads (*). In bottom panel from the left the vertical broken lines represent SNPs in the sense DNA strand as follows: orange change to G (2674 and 2758), green change to A (2264), blue change to C (2153, 2363, 2500) and red change to T (1307). 411(1390) bp insertion is depicted with purple, 2 and 27 bp deletion with dashes (2014 and 2826)

Following PCR, amplicons of these selected targets were sequenced in parallel using the Oxford Nanopore platform described above. This facilitated sequencing of *SAG2* (TGGT1_271050; TGME49_271050), where Type II *T. gondii* showed five nucleotide polymorphisms (SNPs) and a three bp insertion when mapped against the Type I reference genome (Fig. 1 and Supplemental Table S5). Similarly, sequencing of *SAG3* (TGGT1_308020; TGME49_308020) demonstrated 27 SNPs (Fig. 2 and Supplemental Table S6); partial sequencing of *ROP17* (TGGT1_288580; TGME49_288580) identified 29 SNPs (Fig. 3 and Supplemental Table S7); and partial sequencing of *ROP21* (TGGT1_288580; TGME49_288580) revealed eight SNPs, a 411 bp insertion in the first intron, and two and 27 bp deletions in the second and third introns respectively (Fig. 4 and Supplemental Table S8).

### Genotyping of wild *T. gondii* infected wood mice

**Fig. 5:**
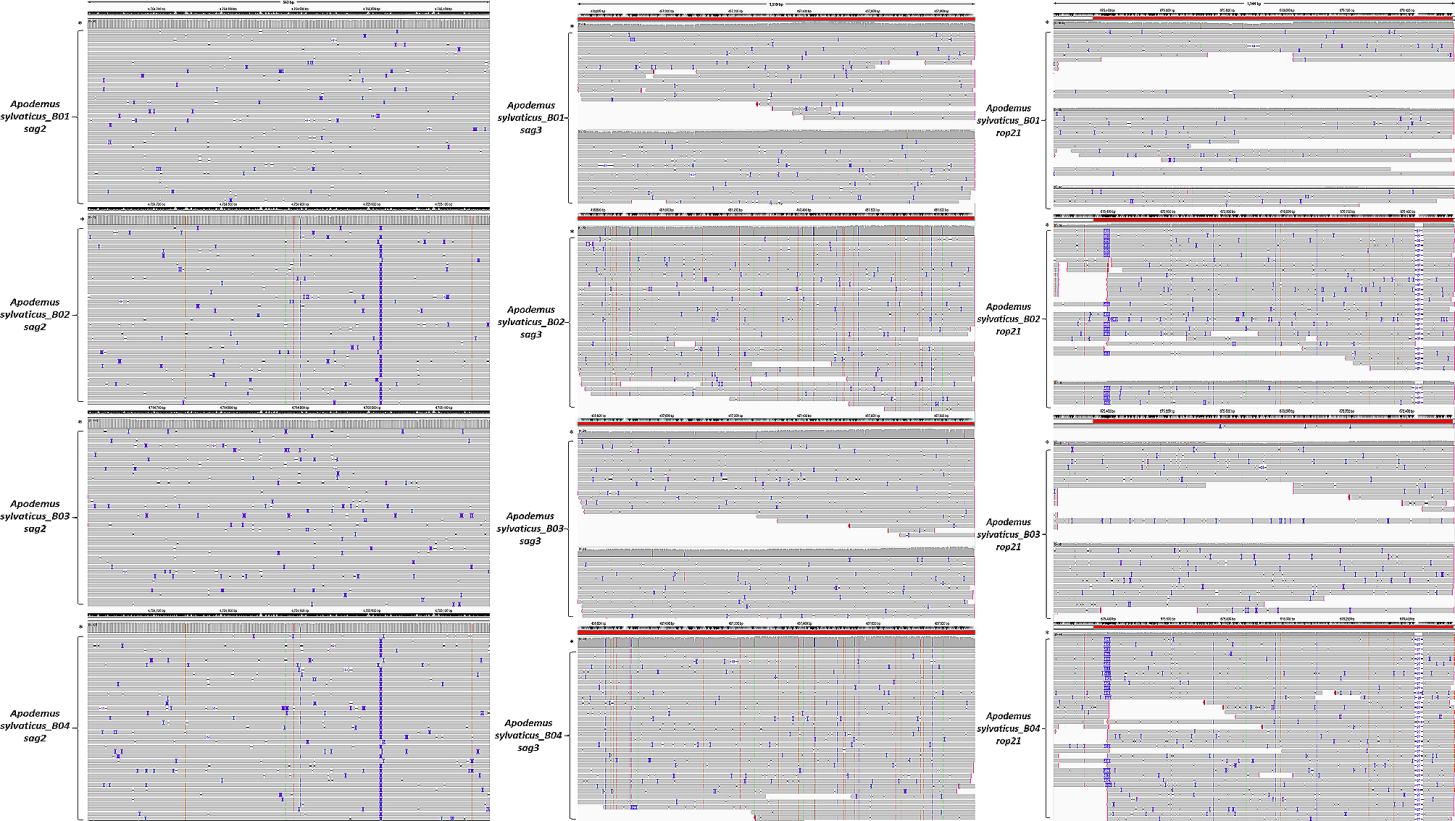
Aligned sequencing reads of the *SAG2*, *SAG3* and *ROP21* genomic loci of four environmental *T. gondii* isolates (B01-B02-B03-B04) as visualised by IVG. Reads were mapped against *T. gondii* Type I genome (TGGT1). Coverage depth > 500x reads (*). In bottom panel from the left the vertical broken lines represent SNPs in the sense DNA strand (*SAG2* and *ROP21*) and antisense (*SAG3*) DNA strand as follows: orange change to G, green change to A, blue change to C and red change to T. Insertions are depicted with purple boxes and deletions with dashes

To assess the utility of the developed Nanopore method in the typing of field samples, *SAG2*, *SAG3* and *ROP21* genomic loci were amplified and sequenced as described from four *T. gondii* samples isolated from individual wild wood mice (*A. sylvaticus*; a kind gift from Professor Geoff Hide, University of Salford). Amplification of the *ROP17* genomic locus did not result in amplicons from any of the animal samples and was therefore excuded from the MLST. The analyses (Fig. 5) revealed that two of the wood mice were infected with *T. gondii* Type I parasites (BO1 and B03) and two with Type II (B02 and B04).

## Discussion

The obligate intracellular parasite *T. gondii* possesses a remarkable mastery to infect a wide range of warm-blooded vertebrates, a characteristic which allows for an almost complete global distribution (Dubey 2021a). Hence, categorization of its genetic heterogeneity is crucial towards furthering our understanding of parasite’s unique infective abilities, distribution and virulence. However, lack of consensus over genotyping methodologies and biomarkers significantly limits our understanding.

*T. gondii* genotyping techniques include restriction fragment length polymorphism (RFLP) (Su et al. 2006), microsatellite (MS) (Blackston et al. 2001; Pomares et al. 2011) and whole or targeted genome sequencing analyses (Joeres et al. 2024; Khan et al. 2007; Sundararaman et al. 2024). This type of information could enable the correlation of variable levels of virulence with different strains of *T. gondii*, identifying and categorizing high-risk and atypical strains so that researchers and clinicians will be able to prioritize preventative measures and improve treatment strategies for vulnerable individuals. Furthermore, genotyping of isolated parasites can shed light on epidemiological understanding and, therefore, potential transmission routes, for example via *T. gondii* reservoirs. Collectively, such genotyping-informed epidemiological analyses will be critical for implementing public health interventions and mitigating future outbreaks through human interventions and animal management.

Utilizing a multi-locus sequence typing (MLST) approach offers a robust and informative methodology for *T. gondii* genotyping. Accuracy and discriminatory power is greatly enhanced compared to PCR-RFLP and MS analyses, providing the potential for a more comprehensive understanding of *T. gondii* genetic diversity and distribution. In this study we have examined the efficiency and efficacy of MLST for the genotyping of *T. gondii* via a portable MinION sequencer (Oxford Nanopore), a method which offers several distinct advantages over other conventional methods, not only in terms of time but also interpretability. We have shown that targeted Nanopore sequencing of specific loci in the *T. gondii* genome yielded sufficient and high-quality data for genotyping within two hours of sequencing runs. Subsequent analyses are easily manageable due to the small volume of data produced. Therefore, the developed platform provided a rapid and robust method to differentiate between Type I and II *T. gondii* that could be adapted to further differentiate between Type I, II, III and other emerging strains (Arranz-Solis et al. 2019).

More precisely we amplified *SAG2* and *SAG3* and fragments of *ROP17* and *ROP21* from *T. gondii* gDNA, two reference strains for Type I and II parasites and four isolates of unknown genotype from the brains of four infected wood mice. *ROP17* amplication of gDNA from the animal sample isolates did not result any product, however genotyping could be based on *SAG2, SAG3* and *ROP21* reads alone. SNPs and InDels in each genomic sequence from reference strains were identified and compared with those found in the animal samples which allowed efficient categorization of the latter as infected with either Type I or II *T. gondii*. Based on the number of the samples and size of fragments, genotyping via this methodology can be completed within two days making the approach rapid and robust.

Targeted MLST demonstrates a clear edge over WGS which is expensive, time consuming and requires powerful computational analyses which many laboratories and reference units do not possess, making it extremely difficult to apply as routine for strain differentiation and typing. Moreover, MLST provides clear-cut data compared to RFLP techniques which rely on variations in fragment sizes, reducing ambiguity whilst simplifying interpretation and analyses. MLST also offers the ability to detect small sequence variations allowing for more detailed differentiation between closely related strains compared to RFLP approaches and methods focused only on single loci. Furthermore, standard sets of genes or genomic loci and sequencing protocols could be adopted to allow data comparisons between different laboratories, promoting collaborations and data sharing. MLST data can also reveal patterns of mutations and recombination events, providing valuable information about how populations evolve and diversify. Furthermore, Nanopore sequencing-based MLST such as we describe here, can be readily multiplexed raising the possibility of adapting this rapid and robust method to detect multiple pathogens within a single sample.

### Electronic supplementary material

Below is the link to the electronic supplementary material.

Supplementary Material 1

## Data Availability

All data supporting the findings of this study are available within the paper and its Supplementary Information. Primer sequences are provided in Supplementary Table S1.
