# Supplementary material for: Rapid genotyping of Toxoplasma gondii isolates via Nanopore-based multi-locus sequencing
Source: AMB Express. 2024 Jun 6;14:68. doi: 10.1186/s13568-024-01728-x (PMC11156620; doi:10.1186/s13568-024-01728-x)
Supplement: Supplementary file 1 — Supplementary Material 1 [file 13568_2024_1728_MOESM1_ESM.pdf]

Rapid genotyping of *Toxoplasma gondii* isolates via Nanopore-based multi-locus sequencing

Zisis Koutsogiannis* and Paul W. Denny*

Department of Biosciences, Durham University, Durham DH1 3LE, UK

*Co-corresponding authors:

zisis.kousogisannis@durham.ac.uk

p.w.denny@durham.ac.uk; Telephone 0191 3343983; orcid.org/0000-0002-5051-1613

Keywords: *Toxoplasma gondii*; genotyping; Oxford Nanopore minION; multi-locus sequence typing (MLST)

Supplementary Information

Table S1: Primer sequences for end-point PCRs

Oligos	Forward	Reverse
SAG2	GAC ACC TTC TGT CTC GTT CCA ATC	GAC CTT TTG ACT ACT TAA TCA TCA GC
SAG3	ATG CAG CTG TGG CGG CGC AGA GCA G	TTA GGC AGC CAC ATG CAC AAG GAG ACC
ROP17	GTC ATT CTC TGA TCG AAA GAC CC	ACT CCT TCT GTA ATA AAG CCG CC
ROP21	CTG CTT CCA GTA ATT TCG TTT TAA TAA AGA	CTG ACA TGC AGG CAC TCT CAG

Table S2: Components and volume of the end-repair/dA tailing ligation reaction

Component	Volume
250 fmol DNA, pooled	50 µL
Ultra II End Prep Enzyme mix	3 µL
Ultra II End Prep Enzyme buffer	7 µL
Total	60 µL

Table S3: Components and volume of the barcoded ligation reaction

Component	Volume
End prepped DNA	22.5 µL
Native barcode (1-12)	2.5 µL
Blunt/TA Ligase master mix	25 µL
Total	50 µL

Table S4: Components and volume of the Oxford Nanopore sequencing adapters ligation reaction

Component	Volume
Pooled barcoded library	67.5 µL
Adapter mix II	2.5 µL
Quick ligation reaction buffer	20 µL
Quick T4 DNA Ligase	10 µL
Total	100 µL

Table S5: Genomic location and analytical documentation of the *Toxoplasma gondii* Type II *SAG2* INDELs and SNPs (chrVIII: 4,754,604 - 4,755,164 [+]) as shown by Nanopore sequencing

Genotype	Chromosome	SNPs and InDels	Position
TypeI/II	chrVIII	T → G	138
TypeI/II	chrVIII	C → A	278
TypeI/II	chrVIII	C → G	289
TypeI/II	chrVIII	G → C	298
TypeI/II	chrVIII	--- → TGG	411 - 413
TypeI/II	chrVIII	A → G	536

Table S6: Genomic location and analytical documentation of the 27 *Toxoplasma gondii* Type II *SAG3* SNPs (chrXII: 456,740 – 457,897 [-]) as shown by Nanopore sequencing

Genotype	Chromosome	SNPs	Position
TypeI/II	chrXII	C → T	1077
TypeI/II	chrXII	A → G	1061
TypeI/II	chrXII	A → C	1053
TypeI/II	chrXII	T → A	1044
TypeI/II	chrXII	T → C	1005
TypeI/II	chrXII	T → A	1001
TypeI/II	chrXII	C → G	981
TypeI/II	chrXII	G → A	792
TypeI/II	chrXII	A → C	685
TypeI/II	chrXII	G → A	643
TypeI/II	chrXII	A → G	573
TypeI/II	chrXII	A → C	514
TypeI/II	chrXII	C → G	513
TypeI/II	chrXII	A → T	501
TypeI/II	chrXII	A → C	468
TypeI/II	chrXII	T → C	466
TypeI/II	chrXII	T → C	401
TypeI/II	chrXII	C → T	323
TypeI/II	chrXII	G → A	319
TypeI/II	chrXII	G → C	351
TypeI/II	chrXII	C → T	238
TypeI/II	chrXII	G → A	231
TypeI/II	chrXII	A → G	216
TypeI/II	chrXII	G → A	159
TypeI/II	chrXII	A → C	150
TypeI/II	chrXII	G → C	125
TypeI/II	chrXII	A → G	93

Table S7: Genomic location and analytical documentation of the 29 *Toxoplasma gondii* Type II *ROP17* SNPs (chrVIIb: 3,287,488 – 3,288,688 [-]) as revealed by Nanopore sequencing

Genotype	Chromosome	SNPs	Position
TypeI/II	chrVIIb	C → G	1569
TypeI/II	chrVIIb	A → G	1554
TypeI/II	chrVIIb	A → C	1573
TypeI/II	chrVIIb	C → G	1486
TypeI/II	chrVIIb	C → G	1483
TypeI/II	chrVIIb	A → G	1482
TypeI/II	chrVIIb	T → G	1447
TypeI/II	chrVIIb	T → A	1417
TypeI/II	chrVIIb	G → A	1292
TypeI/II	chrVIIb	A → G	1290
TypeI/II	chrVIIb	A → T	1247
TypeI/II	chrVIIb	G → A	1236
TypeI/II	chrVIIb	G → A	1196
TypeI/II	chrVIIb	C → T	1193
TypeI/II	chrVIIb	T → A	1142
TypeI/II	chrVIIb	C →T	1121
TypeI/II	chrVIIb	C → T	979
TypeI/II	chrVIIb	G → T	977
TypeI/II	chrVIIb	C → G	968
TypeI/II	chrVIIb	C → T	964
TypeI/II	chrVIIb	G → A	959
TypeI/II	chrVIIb	C → A	955
TypeI/II	chrVIIb	C → A	824
TypeI/II	chrVIIb	T → G	736
TypeI/II	chrVIIb	G → C	721
TypeI/II	chrVIIb	G → C	544
TypeI/II	chrVIIb	G → A	540
TypeI/II	chrVIIb	C → G	528
TypeI/II	chrVIIb	T → C	524

Table S8: Genomic location and analytical documentation of the *Toxoplasma gondii* Type II *ROP21* InDels and SNPs (chrVIIb: 675,115 - 676,556 [+]) as revealed by Nanopore sequencing

Genotype	Chromosome	SNPs and InDels	Position
TypeI/II	chrVIIb	C → T	1307
TypeI/II	chrVIIb	→ +411	1390-1801
TypeI/II	chrVIIb	TT → --	2014-2015
TypeI/II	chrVIIb	T → C	2153
TypeI/II	chrVIIb	C → A	2264
TypeI/II	chrVIIb	A → C	2363
TypeI/II	chrVIIb	T → C	2500
TypeI/II	chrVIIb	C → G	2674
TypeI/II	chrVIIb	C → G	2758
TypeI/II	chrVIIb	→ -27	2826
